# Molecular design and excited state dynamics regulation in phototheranostics

**DOI:** 10.52601/bpr.2025.250004

**Published:** 2025-10-31

**Authors:** Peipei Xing, Xinyue Wu, Mengliang Zhu

**Affiliations:** 1 Beijing Vocational College of Agriculture, Beijing 102442, China; 2 CAS Key Laboratory for Biomedical Effects of Nanomaterials and Nanosafety, National Center for Nanoscience and Technology of China, Beijing 100190, China; 3 University of Chinese Academy of Sciences, Beijing 100049, China

**Keywords:** Phototheranostics, Excited-state dynamics, Photosensitizers, Energy dissipation pathways, Tumor imaging and therapy

## Abstract

Phototheranostic, as an emerging non-invasive cancer diagnosis and treatment modality, combines optical imaging with phototherapy, showcasing advantages such as precision, high efficiency, and low toxicity. The core of phototheranostics lies in photosensitizers (PSs), where molecular design and excited state dynamics regulation are crucial for performance optimization. This review systematically summarizes recent advancements in phototheranostic agents, focusing on strategies for molecular design and their critical role in excited-state energy conversion. Through strategies such as molecular structure optimization, coordination modulation, and self-assembly, the excited-state energy dissipation pathways of phototheranostic agents are precisely regulated, achieving a functional output balance while significantly enhancing therapeutic efficacy. Novel phototheranostic agents integrate multifunctional designs to realize theranostic integration, offering innovative solutions for complex cancer treatment. Finally, this paper explores the development prospects of nanotechnology-based phototheranostic strategies, providing new perspectives and potential breakthroughs for the next generation of phototheranostics.

## INTRODUCTION

**Figure 1:**
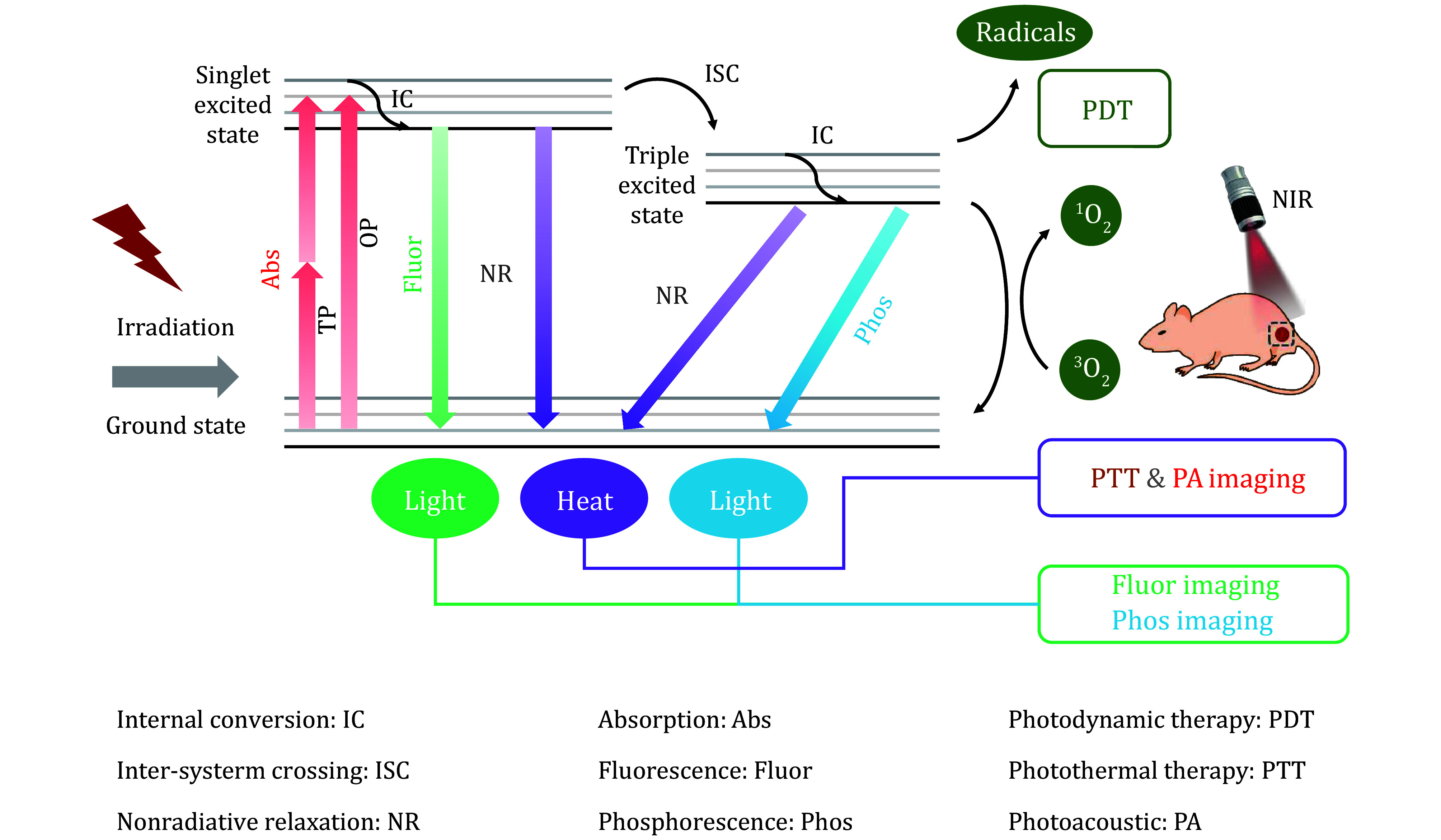
Simplified Jablonski diagram to illustrate various photophysical processes in organic molecules for phototheranostics

Current primary cancer treatments include surgical resection, radiotherapy, and chemotherapy. However, these traditional therapies have significant limitations in terms of treatment efficacy and side effect management, particularly with tumor recurrence and drug resistance (Gu *et al.* 2022). In contrast, phototheranostics, as an emerging non-invasive diagnostic and therapeutic approach, has shown great clinical potential due to its short treatment time, remarkable efficacy, strong anti-resistance properties, and low side effects (Zheng *et al.* 2023). Phototheranostics integrates optical imaging and phototherapy (Yin *et al.* 2022b). Its fundamental principle involves the energy conversion properties of photosensitizers (PSs) upon light excitation, which facilitate cancer cell destruction through multiple energy dissipation pathways while providing real-time imaging for disease diagnosis (Thangudu *et al.* 2021). As illustrated in Fig. 1, the excited-state energy conversion processes of organic molecules can be clearly described using a Jablonski diagram (Feng *et al.* 2020). Under light irradiation, photosensitizer molecules transition from the ground state to the singlet excited state and may subsequently undergo three energy dissipation pathways (Hu *et al.* 2021).

(1) Fluorescence Emission. The singlet excited state undergoes radiative decay to release energy in the form of fluorescence (Dai *et al.* 2021). Near-infrared (NIR) fluorescence imaging technology has been widely utilized in clinical tumor diagnosis due to its strong tissue penetration and low background noise (Cheng and Pu 2020). In recent years, advancements in fluorescence lifetime imaging technology have further enhanced diagnostic precision. By designing suitable NIR fluorescence probes and regulating their excited-state lifetimes, background fluorescence interference can be effectively eliminated, significantly improving the signal-to-noise ratio in imaging (Gao *et al.* 2021).

(2) Photothermal Effect and Photoacoustic Imaging. The singlet excited state undergoes non-radiative decay, releasing energy in the form of heat, and enabling photothermal therapy (PTT) (Lv *et al.* 2022). Upon absorbing light energy, the photosensitizer converts it into heat, leading to a localized temperature increase that can damage cancer cells and induce apoptosis. This treatment modality is oxygen-independent, making it particularly effective in hypoxic tumor environments (Chen *et al.* 2022). Additionally, the thermal effect of the photosensitizer can induce sound wave vibrations, combining optical imaging with ultrasound technology to achieve high-resolution and deep-tissue imaging along with therapeutic functionality (Kyrkou *et al.* 2022).

(3) Photodynamic therapy (PDT). If the singlet excited state transitions to the triplet excited state via intersystem crossing (ISC), the energy can dissipate to the ground state either radiatively or non-radiatively, generating phosphorescence or heat, respectively (Li *et al.* 2022b). The triplet state can also react with oxygen molecules to generate reactive oxygen species (ROS), enabling PDT (Kuzmina *et al.* 2024). PDT leverages ROS to damage cancer cell membranes, proteins, and DNA, inducing apoptosis or necrosis. This approach offers high spatiotemporal resolution with minimal side effects.

The molecular design and regulation of excited-state dynamics play a crucial role in enhancing the efficacy of PSs (Cai *et al.* 2018). The functions and mechanisms of PSs depend not only on their photophysical properties but also on their excited-state dynamic behaviors (Gao *et al.* 2020). By optimizing molecular structure design and controlling excited-state behavior, the performance of PSs can be significantly improved in the following aspects: (1) Light Absorption Efficiency. By introducing conjugated systems or electron donor/acceptor units, the absorption spectrum can be extended into the NIR region, which penetrates deeper into tumors, thus enhancing the absorption efficiency of PSs *in vivo* (Yan *et al.* 2024). (2) Excited-State Yield. The design of PSs must ensure that they possess high excited-state quantum yields, including fluorescence quantum yield and triplet state yield, to achieve more efficient functional output (Zhu *et al.* 2018b). Increasing the fluorescence quantum yield helps enhance imaging signals, while a higher triplet state yield can significantly promote the generation of ROS, thereby increasing the efficiency of PDT. (3) Excited-State Lifetime. A longer excited-state lifetime helps prolong the interaction time between PSs and oxygen or other energy acceptors. Additionally, proper control of lifetimes can prevent premature decay or side reactions, ensuring the effective action of PSs at the tumor site.

The design of modern PSs emphasizes not only single functionality but also the pursuit of multifunctionality (Wang *et al.* 2020). For instance, by regulating the excited state in conjunction with multimodal imaging technologies, integrated diagnosis and treatment can be achieved, resulting in synergistic effects during both the diagnostic and therapeutic processes (Son *et al.* 2022). In summary, through molecular design and excited-state regulation, it is possible to significantly enhance the efficacy of PSs in PDT and PTT while endowing them with multiple functions, thereby further expanding their application potential in cancer diagnosis and treatment. This paper systematically summarizes the latest research progress on phototheranostic agents from the perspective of excited-state dynamics regulation, focusing on the crucial role of molecular design and excited-state energy regulation strategies in phototheranostics. Specifically, we analyze how to precisely modulate the energy dissipation pathways of phototheranostic agents through molecular structure optimization, coordination regulation, and self-assembly strategies, significantly improving their therapeutic efficiency while achieving a balance in functional output. Furthermore, this review provides readers with cutting-edge research insights in the field of photosensitizer design and excited-state regulation and explores future directions for combining nanotechnology to further enhance the therapeutic efficacy and application potential of PSs in phototheranostics.

## MOLECULAR BASIS OF PHOTOTHERANOSTIC AGENTS

### Design principles of phototheranostic agents

The design of phototheranostic agents aims to optimize diagnostic and therapeutic effects while comprehensively balancing the performance of photophysical properties and biological characteristics. Molecular phototheranostic agents have become a key focus of research due to their advantages of easy modification, tunable functionality, good biocompatibility, and rapid metabolism. An ideal phototheranostic agent should possess the following characteristics: a light absorption range that matches the therapeutic light source, especially in the NIR region with stronger tissue penetration (NIR-I: 700–900 nm; NIR-II: 1000–1700 nm); high excited-state quantum yield and optimized excited-state lifetime to enhance the yield of ROS and photodynamic effects; excellent chemical stability and photochemical stability to avoid degradation or photobleaching; high biocompatibility and low toxicity, with targeted modifications to enhance accumulation at the target site, improve therapeutic specificity, and reduce damage to normal tissues (Su *et al.* 2024); furthermore, the integration of imaging, treatment, and multimodal functionalities can achieve more efficient diagnosis and therapy.

To achieve these goals, researchers have developed a series of design strategies to optimize the performance of phototheranostic agents and enhance their therapeutic effects. For example, extending the conjugation of the molecular backbone or introducing strong electron-withdrawing groups can broaden the absorption range of PSs in the NIR region, thus improving tissue penetration; incorporating heavy atom effects or optimizing intramolecular charge transfer (ICT) structures can enhance the excited-state quantum yield and singlet oxygen generation efficiency; designing phototheranostic agents with metal coordination or utilizing the aggregation-induced emission (AIE) effect can optimize the excited-state and triplet-state lifetimes, enhancing the photodynamic effect. Additionally, introducing rigid backbones or using nanoparticle carriers can improve the stability of PSs, reducing the risk of photobleaching and degradation; adding hydrophilic groups, biological targeting ligands, or smart-responsive units further enhances the biocompatibility and tumor targeting of phototheranostic agents (Liu *et al.* 2021). Notably, multifunctional design can integrate imaging and therapeutic functions, achieving a comprehensive approach to diagnosis and treatment, thereby better meeting the needs of precision medicine. These systematic strategies, from molecular regulation to functional integration, lay a solid foundation for the development of efficient and safe phototheranostic agents.

### Comparison of classic PSs and novel phototheranostic agents

Phototheranostic agents are core tools that utilize light energy to treat diseases, encompassing two major categories: classic PSs and novel phototheranostic agents. Traditional PSs refer to porphyrins and phthalocyanines that have been used clinically or preclinically, such as Hematoporphyrin Derivative (HpD), Temoporfin (mTHPC), and Silicon Phthalocyanine (Pc 4), which are conjugated macrocyclic molecules that have been widely used in PDT due to their excellent photophysical properties and good biocompatibility. Under specific wavelength irradiation, these molecules can generate singlet oxygen, effectively killing tumor cells. Furthermore, these molecules can achieve a balanced distribution between singlet and triplet states, enabling them to produce ROS and allowing imaging through fluorescence emission, making them commonly used in fluorescence imaging-guided diagnosis and treatment (Chen *et al.* 2023a).

However, with advancements in technology and increasing clinical demands, the performance metrics of phototheranostic agents continue to improve, leading to higher requirements for their light absorption range, excited-state quantum yield, biocompatibility, and targeting capabilities. Novel phototheranostic agents have emerged, including porphyrin-like compounds with chemical modifications to enhance their photophysical properties, dye-based PSs, and organic semiconductor polymers. These structurally modified or newly designed compounds also exhibit enhanced properties, such as broader light absorption ranges, higher excited-state efficiencies, and multimodal capabilities, allowing them to integrate multimodal imaging and unified diagnostic and therapeutic functions (Du *et al.* 2023).

In comparison to classic PSs, novel agents demonstrate significant advantages in complex application scenarios, driving the development of phototheranostic technologies. The following sections will further explore the classification, characteristics, and comparison of classic PSs and novel phototheranostic agents, focusing on the advantages and limitations of each.

#### Porphyrinoids

Porphyrinoids are extensions and derivatives of porphyrin structures, exhibiting significant differences in structure and properties compared to traditional porphyrins and phthalocyanines. Traditional porphyrins and phthalocyanines are characterized by high-symmetry tetra-pyrrole macrocycles that possess excellent photosensitizing properties and biocompatibility (Luo *et al.* 2021); however, their absorption spectra are primarily concentrated in the visible light region, which somewhat limits their application in deep tissue therapy. Porphyrinoids significantly enhance photophysical performance through partial hydrogenation, introduction of new aromatic substituents, or expansion of conjugated systems, injecting new vitality into phototheranostic technologies (Sarbadhikary *et al.* 2021).

For instance, benzo-porphyrins enhance the photochemical stability of the molecule and significantly extend light absorption into the NIR region by incorporating benzene rings into the conjugated system of the porphyrin macrocycle, providing better options for deep tissue imaging and phototherapy (Zhang *et al.* 2020a). Porphyrazine, by replacing *meso* carbon atoms in the porphyrin ring with nitrogen atoms, forms highly symmetrical aromatic systems that exhibit excellent light stability and high absorption coefficients, making them important candidates for photocatalysis and PDT. Additionally, expanded porphyrins-macrocyclic architectures featuring extended conjugation through additional pyrrole units or expanded ring sizes (*e*.*g*., sapphyrins with five pyrrole subunits and texaphyrins with six-membered macrocycles)-exhibit enhanced NIR absorption capabilities through the formation of larger conjugated π-systems (Pareek *et al.* 2012). This structural modification enables broad applications in NIR-mediated PDT and PTT, and photoacoustic imaging. Furthermore, multimodal theranostic porphyrin derivatives are increasingly designed through strategic incorporation of functional moieties, such as chelating agents (DOTA, NOTA, or DTPA) for lanthanide metal coordination (Ning *et al.* 2019), or covalent conjugation to targeting ligands (folic acid or peptides) (Hamley *et al.* 2017). These hybrid architectures enable simultaneous tumor targeting, real-time imaging (via MRI, fluorescence, or nuclear modalities), and light-activated therapeutic functions within a single molecular platform.

Porpholactones are typical porphyrinoids. The introduction of a carbonyl substituent at the β position of the porphyrin ring significantly reduces the aromaticity of the macrocycle, forming unsaturated porphyrin derivatives (Xu *et al.* 2021). By introducing lactone groups and regulating regional isomer effects, the NIR light absorption capacity of porpholactone is significantly enhanced, making them ideal PSs for PDT and NIR imaging (Ning *et al.* 2019). Importantly, this structural adjustment allows for precise control of the energy transfer processes of singlet oxygen sensitization, photocatalytic reactions, and lanthanides NIR luminescence, thus meeting different biological functional needs. Furthermore, porpholactones possess excellent chemical stability and biocompatibility and have been widely applied in tumor treatment, catalytic chemistry, and related fields. Similarly, chlorophyll-like compounds enhance their aromaticity by partially hydrogenating the porphyrin ring, resulting in a significant redshift of their absorption spectra, making them particularly suitable for deep tissue phototheranostic applications (Zhu *et al.* 2022). These compounds not only exhibit excellent photosensitizing properties but can also coordinate with transition metals, mimicking the functions of natural metal enzymes, thereby deepening the understanding of photoreaction mechanisms and playing a crucial role in biomimetic catalysis.

These porphyrinoids, with innovative structural designs and diverse functionalities, significantly surpass traditional porphyrins, showcasing their immense potential in the field of phototheranostics and providing new directions for further exploration of multifunctional PSs.

#### Cyanines

Cyanines are a class of molecules characterized by conjugated polyene chains, with their molecular backbone typically consisting of two nitrogen-containing heterocyclic cations connected by π-conjugated bridges, forming a linear conjugated system (Bilici *et al.* 2021). Their typical azo bonds (N=N) or imine bonds endow the dyes with powerful spectral regulation capabilities, allowing precise modulation of absorption and emission wavelengths through simple molecular modifications. Compared to traditional photosensitive molecules, cyanines have larger conjugated systems, and their absorption spectra can extend into the NIR region, enabling efficient optical diagnosis and treatment of deep tissues (Liu *et al.* 2024).

Cyanines exhibit outstanding features, including high molar extinction coefficients (ca. 10^5^ (mol/L)^-1^·cm^-1^) and good fluorescence quantum yields, making them excellent candidates in fields such as fluorescence imaging and PTT/photoacoustic imaging (Zhao *et al.* 2024). Representative cyanine molecules include Indocyanine Green (ICG) and dyes such as Cy5 and Cy7. Among these, ICG which has a molar extinction coefficient of 1.7 × 10^5^ (mol/L)^-1^·cm^-1^ at its absorption maximum, has been approved for clinical imaging and PTT, demonstrating good safety and efficacy. However, cyanines still face several challenges in practical applications, such as insufficient photostability and molecular stability issues in complex biological environments. In aqueous solutions, cyanine molecules are prone to intermolecular aggregation, leading to fluorescence quenching and uneven biodistribution. Insufficient photostability is also a significant drawback. In the blood circulation and under light exposure, the structure of cyanine dyes is easily oxidized and degraded, significantly affecting their biocompatibility and therapeutic efficiency. Furthermore, the conjugated systems in cyanine molecules are relatively sensitive to complex biological environments, which may result in decreased molecular stability. Strategies like encapsulation into nanoparticles or conjugation with polyethylene glycol (PEG) have shown promise in improving the water solubility and photostability of cyanine dyes (Zhu *et al.* 2018d).

To address these issues, researchers have proposed various strategies to optimize the performance of cyanines. One effective method is to introduce hydrophilic groups, enhancing water solubility through chemical modification to avoid aggregation-induced fluorescence quenching. For example, incorporating sulfonic acid groups, carboxylic acid groups, or polyethylene glycol chains into the molecular structure not only significantly improves water solubility but also enhances the stability of the dyes in biological environments. Additionally, combining cyanines with nano-carriers is an important approach to enhance their performance. Nanomaterials such as liposomes, polymer nanoparticles, and porous silicon can protect cyanine dyes from oxidation and photodegradation while improving drug delivery efficiency, thus enhancing their therapeutic effects *in vivo*.

#### Organic semiconductor polymers

Organic semiconductor polymers are a class of functional materials based on conjugated polymer backbones, garnering significant attention in the field of phototherapy due to their excellent optical and electronic properties (Yin *et al.* 2021). These polymers form extended π-conjugated systems through the alternating arrangement of electron donor and acceptor units, endowing them with unique advantages such as broad spectral absorption, strong NIR light response, and high photothermal conversion efficiency. Compared to traditional small-molecule PSs, organic semiconductor polymers exhibit significant differences in molecular structure and photoelectric performance, presenting new opportunities for PDT and PTT.

The light absorption spectra of organic semiconductor polymers typically cover the entire visible to NIR regions, particularly showing strong absorption characteristics in the NIR-II region, which provides unique advantages in PTT and photoacoustic imaging of deep tissues. For example, classic polymers like P3HT (poly(3-hexylthiophene)) and PDPP3T (a polymer based on dibenzothiophene) have been widely used in research on PTT, photoacoustic imaging, and multimodal theranostic. These polymers not only exhibit efficient photothermal conversion but can also be further optimized in terms of excited-state regulation through molecular design, thereby enhancing therapeutic efficacy.

Semiconductor polymers designed with donor-acceptor (D-A) structures have garnered particular attention (Niu *et al.* 2024). This type of structure allows for effective modulation of optical performance through molecular design, enhancing the generation efficiency of singlet oxygen and photoelectric conversion performance. By encapsulating high-quality D-A systems in amphiphilic compound F127, semiconductor polymer nanoparticles can be successfully prepared. These nanoparticles exhibit good water solubility and biocompatibility while maintaining their original photophysical properties.

## EXCITED-STATE DYNAMICS REGULATION

### Excited-state properties

The excited state is the condition of a molecule after it absorbs light energy and transitions from the ground state to a higher energy level. The properties of the excited state play a crucial role in the efficacy of photosensitive molecules in phototherapy. The main forms of excited states include singlet states and triplet states, and their differences determine the behavior of the molecule in the excited state, energy dissipation mechanisms, and interactions with the surrounding environment (Ortiz-Rodriguez *et al.* 2021).

The singlet state is a common form of excited state, where the electrons in the molecule have antiparallel spins, resulting in higher energy and stronger spin symmetry. The singlet state can return to the ground state through radiative transitions or non-radiative transitions. Among these, fluorescence is a way for excited-state molecules to release energy through photons; although common, its quantum efficiency is usually low. Internal conversion involves transitions between energy levels due to molecular vibrations and rotations, converting excited-state energy into thermal energy. Internal conversion is one of the core mechanisms of PTT, where the molecule absorbs light energy and converts it into heat, leading to an increase in local temperature and subsequently inducing cell death.

The triplet state is another important form of excited state, where the electrons in the molecule have parallel spins, resulting in lower energy and weaker spin symmetry, thus having a longer lifetime. The triplet state returns to the ground state through radiative transitions, a process that is typically slower and produces weaker light. Additionally, the triplet state can engage in electronic or energy transfer with molecular oxygen, generating ROS. This process is a core mechanism of PDT. ROS possess strong oxidative properties and can cause oxidative damage to cells and tissues, inducing apoptosis and achieving therapeutic effects.

### Regulation strategies for excited-state dynamics and energy dissipation

Excited-state dynamics are crucial in the design and application of phototherapeutic agents, as their lifetime, energy transfer efficiency, and energy dissipation pathways directly impact the functionality and efficacy of these agents. To enhance the performance and application range of phototherapeutic agents, researchers employ various design strategies to regulate excited-state dynamics, including structural modification, coordination regulation, and self-assembly. By optimizing photophysical processes such as light energy absorption, energy transfer, and energy dissipation, these strategies significantly improve the optical performance and therapeutic effects of phototherapeutic agents while achieving a balance of different therapeutic functions.

Structural modification of the molecule is a common method for regulating excited-state dynamics. By adjusting the chemical structure of the photosensitizer, the energy level distribution, electron density, and optical properties can be tuned. For example, introducing or modifying electron donor and acceptor groups can effectively alter the excited-state lifetime, energy transfer efficiency, and fluorescence quantum yield, thereby optimizing therapeutic performance. Coordination regulation optimizes excited-state dynamics by fine-tuning the interactions between the PS and metal ions or other molecules, thereby modulating electronic transition processes to control the excited-state lifetime and energy dissipation pathways. Self-assembly is a highly flexible and effective strategy. By self-assembling photosensitizer molecules into nanostructures or aggregates, the excited-state dynamics can be significantly improved, enhancing their therapeutic effects. These regulation strategies not only enhance the photodynamic and photothermal performance of PSs but also provide strong support for the multifunctionalization of phototherapeutic agents.

#### Molecular structural modification

Structural modification is one of the core methods for regulating the excited-state dynamics of photosensitive molecules. By precisely designing and modifying molecular structures, the properties of the excited states can be effectively adjusted, influencing energy transfer efficiency, excited-state lifetime, and the transition pathways between the ground state and the excited state. In the molecular design of PSs, expanding the conjugated system is a common and effective approach. The conjugated system provides a stable electron cloud distribution, reduces energy loss in the excited state, and facilitates effective electron transfer. By extending the conjugated system, the light absorption range of the photosensitizer can be expanded from the visible region to the NIR region, enhancing its penetration ability in biological tissues.

**Figure 2:**
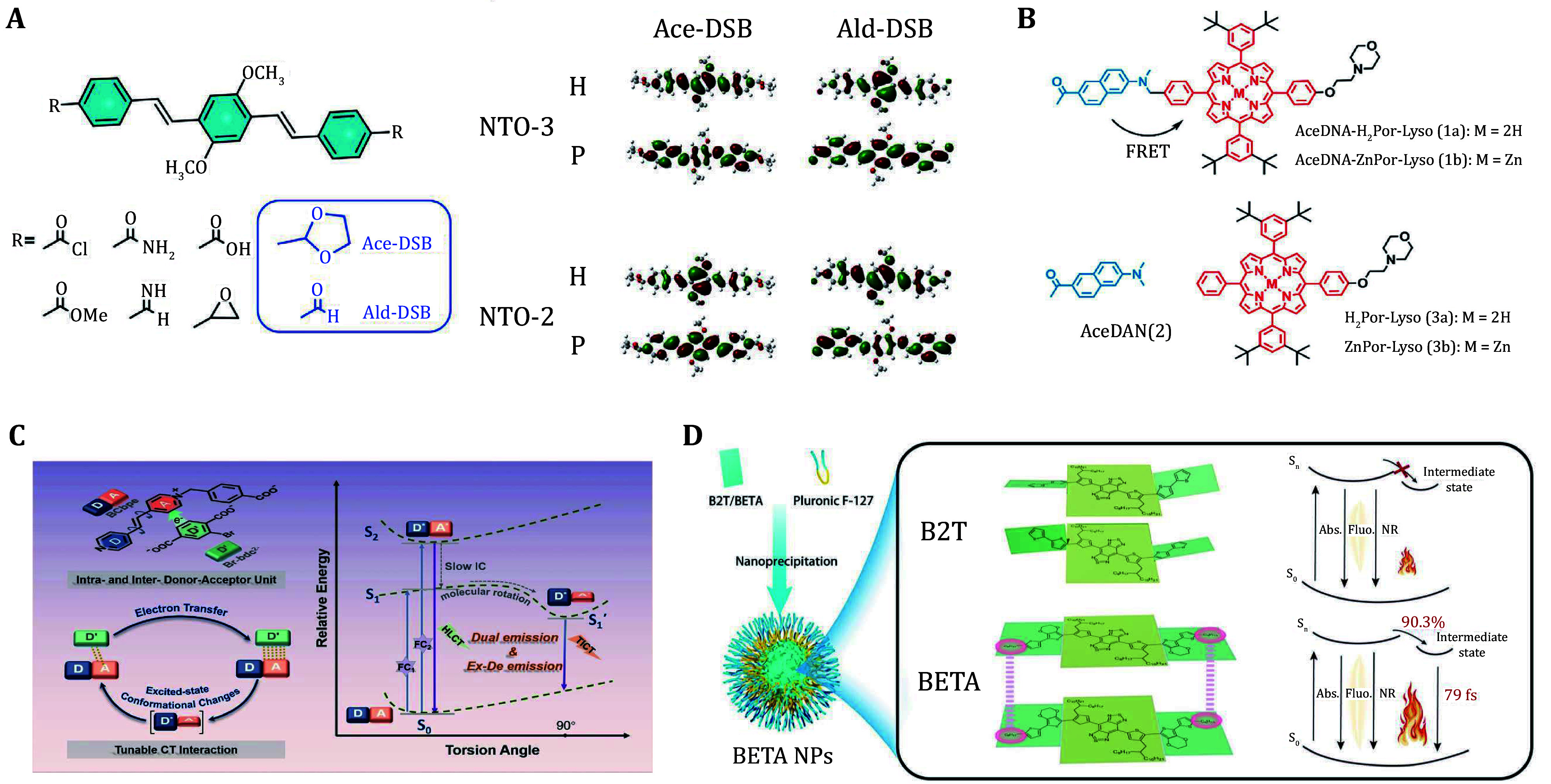
**A** The DSB derivatives designed for initial screening and NTOs of S2, S3 for Ald-DSB and Ace-DSB related to the TPA process (Sun *et al*. 2019). **B** Structures of two-photon dyad AceDAN-H_2_Por-Lyso (Zhu *et al*. 2018c). **C** Schematic illustration of photophysical and photochemical processes of BCbpe unit and related conformational variation (Pan *et al*. 2025). **D** Schematic of the design principle of D-A-D-type NIR-II-absorbing CSM (Chen *et al.* 2023b)

Two-photon absorption (TPA), as a nonlinear optical phenomenon, has demonstrated enormous potential in fields such as biological imaging and PDT in recent years, especially in achieving deep tissue penetration, low phototoxicity, and high spatial resolution using NIR light. By optimizing molecular structures, such as expanding the π-conjugated system and constructing push-pull electronic structures, the dynamics of excited states can be effectively modulated, significantly enhancing the TPA cross-section. These structural modifications not only enhance the two-photon absorption capabilities of PSs but also allow precise control over excited-state lifetime and energy transfer efficiency. Sun *et al*. proposed a theoretically assisted molecular screening method for designing TPA dyes capable of effectively generating two-photon excited fluorescence and singlet oxygen (Fig. 2A). This study optimized the excited-state characteristics by adjusting the molecular structures of diphenyl ethylene (DSB) derivatives. By calculating the transition dipole moments and natural transition orbitals (NTO) of the excited states, a relationship between molecular structure and excited-state characteristics was established (Sun *et al.* 2019). The transformation from the acetaldehyde terminal structure (ACE-DSB) to the aldehyde terminal structure (ALD-DSB) significantly enhanced the TPA cross-section and improved the efficiency of singlet oxygen generation, reducing phototoxicity to normal tissues and thus enhancing imaging capabilities and the effectiveness of PDT.

Fluorescence resonance energy transfer (FRET) is a strategy to regulate energy dissipation pathways through non-radiative energy transfer between excited-state molecules (Zhang *et al.* 2020b). In FRET design, molecules with strong emission energy (donors) are combined with molecules that can accept energy (acceptors). When the donor molecule is excited, the excited-state energy is transferred to the acceptor molecule via the FRET mechanism, thereby regulating the distribution of excited-state energy and improving the reaction efficiency of the photosensitizer (Zhu *et al.* 2018a). Zhu *et al*. developed a two-photon-excited fluorescent dye, Acedan-H_2_por-LYSO dyad for targeted lysosomal imaging and PDT of cancer cells using this indirect excitation design strategy (Fig. 2B). This dyad achieves two-photon excited fluorescence imaging and PDT treatment through the FRET mechanism (Zhu *et al.* 2018c). In this system, the AceDan part acts as the donor for two-photon absorption, while the porphyrin(Zn) serves as the energy acceptor. After two-photon excitation, the excited-state energy of the AceDan donor is effectively transferred to the porphyrin(Zn) acceptor via FRET, achieving a transfer efficiency of 98% and generating red fluorescence and singlet oxygen. This study highlights the optimization of the excitation process, enabling deeper tissue penetration and reducing side effects, providing an efficient and low-toxicity strategy for cancer imaging and treatment.

In the research of regulating the excited-state properties of photosensitive molecules, the design of donor-acceptor (D-A) conjugated structures has always been considered one of the classic strategies for enhancing molecular performance (Feng *et al.* 2022). By constructing conjugated systems with push-pull electronic effects, not only can the optical and excited-state dynamics properties of the molecules be significantly optimized, but their performance can also be precisely regulated according to application needs (Li *et al.* 2022a). For example, the D-A structured semiconductor polymer (SP) designed by Li *et al*. exhibited an exceptionally high quantum yield of singlet oxygen, primarily benefiting from a low energy gap (1.68 eV) that enhanced the ISC efficiency. Through this design, they achieved efficient capture of NIR light, facilitating effective imaging and ablation of cancer cells in PDT. Meanwhile, the dynamic adjustment of molecular structures to further optimize the excited-state properties of the D-A system has also attracted significant attention. Pan *et al*. introduced a rotatable C-C single bond into the donor-acceptor pair, utilizing photomodulation to regulate the charge transfer (CT) process, thereby achieving dynamic control of the excited-state conformational changes (Fig. 2C). This design not only allows for the activation and deactivation of CT interactions but also provides feedback signals for dual-emission imaging, bringing more possibilities for integrated diagnostic and therapeutic applications (Pan *et al.* 2025).

Additionally, enhancing the planarity and light absorption capability of molecules by introducing aromatic rings or specific substituents can significantly improve excited-state behavior. The research by Chen *et al*. optimized the NIR-II absorption and photothermal properties of conjugated small molecules by adjusting the donor and side chain design (Chen *et al.* 2023b). This strategy improved the photothermal conversion efficiency of the molecules and developed bisthiadiazole-ethoxylene-thiophene-alkyl chain (BETA) nanoparticles suitable for NIR-II imaging and treatment, further advancing the development of PTT (Fig. 2D). For the molecular design of photothermal therapeutic agents, Liu et al. proposed a strategy to enhance performance by improving the motion of polymer molecules. By grafting molecular rotors and long alkyl chains onto the D-A core, they not only reduced intermolecular interactions but also enhanced non-radiative decay efficiency by forming twisted intramolecular charge transfer states (TICT) (Liu *et al.* 2019). The NIRb14 nanoparticles developed using this method exhibited excellent photothermal performance, enabling precise PTT guided by photoacoustic imaging.

#### Coordination strategy

In addition to molecular structural modification, the coordination strategy serves as an important means of regulating the excited-state dynamics of phototherapy agents, offering unique advantages (Liu *et al.* 2023). By introducing specific metal centers and finely designing ligand structures, the excited-state energy distribution, lifetime, and electron transfer efficiency of photosensitive molecules can be significantly optimized. This strategy not only enhances the photophysical and photochemical properties of photosensitive molecules but also imparts multifunctionality (Imberti *et al.* 2020). For example, the introduction of Gd ions can endow phototherapy agents with magnetic resonance imaging (MRI) capabilities (Li *et al.* 2022c), while the addition of Fe ions can induce ferroptosis to achieve synergistic tumor therapy, thus demonstrating higher efficiency and broader application potential in both imaging and treatment (Zhang *et al.* 2023).

**Figure 3:**
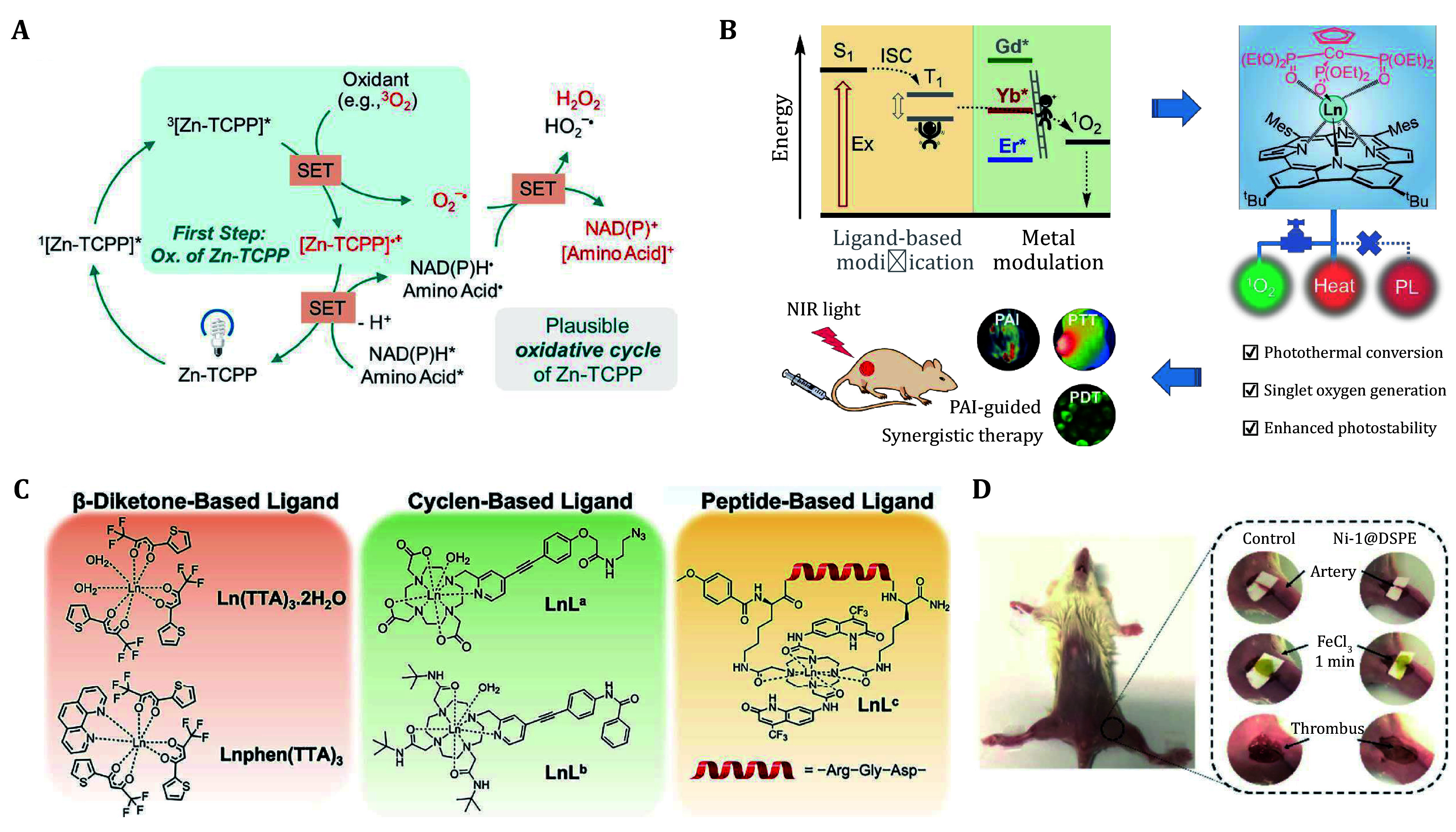
**A** Plausible oxidative cycle of Zn-TCPP involving NADH, NADPH, and some amino acid oxidation (Yu *et al*. 2023). **B** Schematic illustration of the energy dissipation pathways in LnL complexes and the proposed phototheranostic (Zhu *et al*. 2021). **C** The chemical structures of β-diketone-based, cyclen-based, and peptide-based ligands coordinate with Ln ions (Thor *et al*. 2024). **D** *In vivo* thrombus treatment with different concentrations of Ni-1@DSPE under 785 nm laser irradiation (Yao *in vivi* 2022)

Ligand modulation significantly affects the electron cloud density and coordination environment (such as electronic effects and spatial configuration) of metal ions through precise adjustments of ligand structures, thereby effectively regulating the energy distribution and lifetime of excited states (Kitagawa *et al.* 2023). Additionally, the choice of different metal ions (such as Zn^2+^, Cu^2+^, Fe^3+^, *etc*.) can also significantly alter the interactions between the ligands and metal centers, further affecting the properties and energy transfer patterns of the excited states. For example, Zn complexes typically exhibit good luminescent properties, high stability, and excellent ROS generation capabilities in the excited state, effectively promoting the generation of singlet oxygen while achieving integrated diagnosis and therapy. Yu *et al*. significantly enhanced the photocatalytic activity of Zn-TCPP in live cells (Yu *et al.* 2023). The mechanism of Zn-TCPP involves an oxidative cycle, where Zn-TCPP donates electrons to oxygen molecules under light irradiation, generating superoxide anion radicals. The introduction of the zinc center increased the rate of ISC, thereby promoting effective triplet state formation and enhancing photocatalytic performance. During the catalytic cycle, Zn-TCPP can oxidize NADH, NADPH, and specific amino acids, particularly methionine, while generating hydrogen peroxide as a byproduct (Fig. 3A). This mechanism demonstrates the unique role of metal ions in enhancing photocatalytic performance and shows good application prospects in catalytic antitumor therapy for glioblastoma.

In metal porphyrin-based photosensitive systems, the metal center plays a crucial role, with lanthanide elements (Ln) characterized by the formation of stable trivalent ions and having the electronic configuration of [Xe]4f^n^ (*n* = 0–14). The photophysical properties of these elements are characterized by partially filled 4f orbitals, resulting in unique optical behaviors and energy level structures. Different lanthanide metal ions directly influence the electronic structure and excited-state dynamics of the complexes. The combination of rare earth metal ions with classic PSs has been shown to modulate energy transfer dynamics, thereby improving imaging quality and therapeutic efficacy. Zhu *et al*. proposed a metal modulation strategy to adjust the photophysical properties of rare earth metal PSs (Ln-PSs) used in cancer phototherapy (Fig. 3B). Ln metals (such as Gd, Yb, and Er) typically exist stably as trivalent cations, featuring unique 4f inner electron configurations and rich electronic energy levels that present a "staircase" level distribution when mixed with ligand excited state energy levels (Zhu *et al.* 2021). This characteristic makes Ln metals ideal choices for regulating the energy dissipation pathways of excited states. By substituting Ln ions, the integration and balance of different functions within phototherapy molecules can be achieved. Based on NIR absorbing carbazole porphyrin ligands, a series of complexes with different rare earth metal centers were synthesized. These complexes exhibited strong absorption in the 650–850 nm therapeutic window and demonstrated high photothermal conversion efficiency and singlet oxygen generation capability. Importantly, the energy dissipation pathways and the ability of Ln complexes to sensitize singlet oxygen under light excitation highly depend on the energy gap between the ligand triplet state and the rare earth ion excited state. A smaller energy gap promotes effective energy transfer and stabilizes the excited state, while a lower excited state of rare earth ions can lead to the loss of the system's ability to sensitize oxygen, transferring energy to the Er ions. YbL exhibited a balance between singlet oxygen generation and thermal deactivation, making them suitable for combined imaging and treatment applications. In a mouse tumor model, YbL@MSN demonstrated the ability to achieve photothermal/photodynamic synergistic therapy guided by photoacoustic imaging *in vivo*, significantly inhibiting tumor growth after a single NIR laser irradiation, thereby validating the effectiveness of this strategy in practical applications.

Thor *et al*. studied the energy transfer dynamics of organic Ln compounds, revealing that energy transfers from the triplet state to the nearest Ln ion energy level, rather than directly to the emissive state (Thor *et al.* 2024). The research indicated that the design of ligands can influence the energy transfer pathway, thereby optimizing the transition from the triplet state to the nearest Ln ion energy level. To optimize light harvesting, the energy gap between the triplet state and the nearest receiving state should be adjusted. Furthermore, the ISC rate, affected by molecular structure, plays a significant role in the excited state dynamics and luminescence of Ln ions (Fig. 3C).

By designing ligand molecules that can modulate the interactions between ligands and metal ions, the excited state properties of PSs can be significantly impacted. This regulatory mechanism enables PSs to exhibit higher performance in photophysical and photochemical applications, particularly in enhancing the photothermal conversion efficiency and improving excited state stability. Yao *et al*. designed a series of non-aromatic benzothiophene macrocyclic nickel (II) complexes that combine PTT and photoacoustic imaging (Yao *et al.* 2022). Specifically, the mechanism of the Ni-1 complex involves the formation of strong α-donating Ni-C bonds, which induce a redshift of the absorption band into the NIR region, thereby enhancing its photothermal properties. After photoexcitation, Ni-1 fills the ligand center and three MLCT states, facilitating efficient photothermal conversion. Femtosecond transient absorption studies revealed characteristics of the excited state dynamics, indicating the decay of ground state bleaching and the formation of new absorption bands, reflecting the vibrational relaxation process. Encapsulating Ni-1 in nanoparticles (Ni-1@DSPE) further modulated its excited state dynamics, achieving effective photothermal conversion and allowing its application in thrombus treatment through photoacoustic imaging (Fig. 3D). The application of coordination strategies, particularly the design of ligands and the selection of coordinating metals, plays a critical role in defining excited state properties and energy dissipation processes, directly affecting the effectiveness of imaging and therapeutic functions.

#### Molecular self-assembly

Molecular self-assembly technology utilizes intermolecular interactions (such as hydrogen bonding, π-π stacking, electrostatic interactions, *etc*.) to spontaneously organize photosensitizer molecules into nanomaterials with specific stacking structures (Perez-Castillo *et al.* 2024). These self-assembled materials not only enhance the stability of PSs but also regulate their excited state dynamics, improving therapeutic efficacy. The aggregation behavior of molecules during the self-assembly process significantly impacts their excited state dynamics, particularly in controlling luminescent properties and energy transfer pathways (Yao *et al.* 2025). The AIE effect is a key phenomenon where certain molecules exhibit stronger optical performance in their aggregated state than in their monomer state (Yin *et al.* 2022a). By forming nanoparticles or supramolecular structures through self-assembly, the luminescent efficiency of molecules in the solid or aggregated state can be significantly enhanced, and the excited state lifetime can be extended, optimizing energy dissipation pathways. This effect has important application value in the optimization of the photophysical properties of PSs.

**Figure 4:**
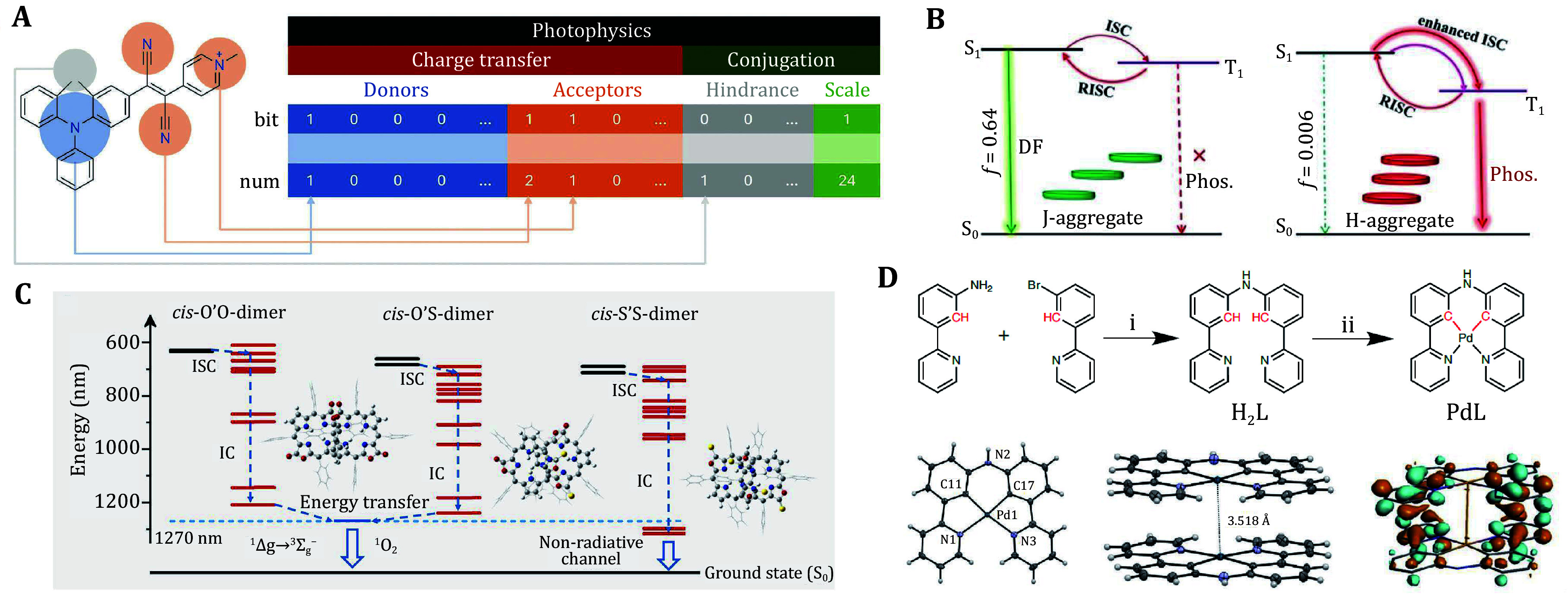
**A** Schematic view of generating a photophysics-oriented fingerprint (Gong *et al*. 2024). **B** Energy level diagrams for g-crystal and r-crystal emissions (Li *et al*. 2021). **C** Depiction of the proposed excited state energy dissipation pathways and triplet energy levels of porphothiodilactones (Zhu *et al*. 2024). **D** Synthesis, crystalline structure, and DFT calculation of LUMOs of PdL as a dimer (Zhou *et al*. 2023)

Gong *et al*. utilized machine learning techniques for molecular design in AIE systems (Gong *et al.* 2024), proposing a novel chemical fingerprint called POFP for classifying and predicting the photophysical properties of organic materials (Fig. 4A). The study indicated that the interactions between donor and acceptor components and the conjugated structure of the molecules significantly influence the emission wavelength and optical performance. The non-planar geometries in AIE systems enhance luminescence intensity in the condensed state by suppressing exciton diffusion and quenching effects. In contrast, under dilute conditions, the non-adiabatic coupling between the excited state and ground state leads to enhanced molecular motion, promoting non-radiative decay and reducing emission intensity. This indicates that the interaction between molecular structure and excited state properties is a core factor influencing the AIE effect. Fujimoto *et al*. revealed the role of molecular structural changes on excited state dynamics through the study of the AIE properties of diphenylmethane boron difluoride complexes (Fujimoto *et al.* 2024). In particular, restricting intramolecular motion in solid-state molecules effectively suppresses non-radiative transitions, significantly enhancing the emission characteristics of AIE compounds.

Aggregation behavior plays a crucial role in the dynamics of molecular excited states and the regulation of photophysical properties, particularly in optimizing thermally activated delayed fluorescence (TADF) and room-temperature phosphorescence (RTP) emissions. Li *et al*. studied the mechanism of regulating molecular luminescent properties through J and H aggregation, finding that J aggregation facilitates ultrafast reverse ISC (RISC) by narrowing the energy gap between the lowest singlet state and triplet state, thereby enhancing radiative singlet decay and ultimately achieving TADF enhancement (Li *et al.* 2021). In contrast, H aggregation accelerates ISC while suppressing radiative decay, stabilizing triplet excitons and supporting RTP emission (Fig. 4B). This synergistic effect between the aggregated structure and excited state dynamics is key to regulating the performance of organic luminescent materials. Fukaya *et al*. revealed the role of J-type exciton coupling in the formation of sheet-like aggregates by studying the self-assembly of amide-functionalized thienyl diketopyrrolopyrrole (TDPP) dyes in aqueous media (Fukaya *et al.* 2022). The aggregates exhibited more efficient exciton diffusion and coherent vibrations, and a quantitative assessment of exciton-exciton annihilation and singlet fission processes further demonstrated the impact of the aggregated structure on excited state properties.

Zhu *et al*. investigated the effect of introducing sulfur heteroatoms into porphyrin derivatives, noting that increased sulfur substitution can enhance ISC and the generation of ROS; however, in aqueous solutions, it may undergo aggregation-induced deactivation (Zhu *et al.* 2024). Molecular dynamics simulations and time-dependent density functional theory calculations were employed to understand the aggregation and excited state dynamics. The mechanism involves progressive sulfurization leading to the formation of J aggregates, which affects the energy levels of the triplet states. When the triplet state energy level is lower than the excited state energy level of singlet oxygen, the energy dissipation pathway is altered, shifting from sensitizing oxygen to non-radiative relaxation of the triplet state (Fig. 4C). These studies indicate that by regulating molecular self-assembly and aggregation behavior, one can significantly influence the excited state dynamics, thereby optimizing the performance of molecules in imaging and phototherapy, further advancing the applications of organic materials in biomedical and optoelectronic fields.

Self-assembled nanomaterials have played an important role in targeted delivery applications within photosensitizer systems, significantly enhancing the accumulation efficiency of PSs in target tissues while reducing their non-specific distribution *in vivo*, thereby improving therapeutic efficacy. Zhou *et al*. designed a self-assembling molecular drug — a ring-metallated palladium complex (PdL) — which exhibited excellent tumor-targeting capabilities and extended circulation time *in vivo* (Fig. 4D). These nanoparticles not only have low systemic toxicity but also demonstrate high efficacy in PDT (Zhou *et al.* 2023). Upon light activation, PdL effectively inhibited tumor growth, maintaining stable photodynamic properties even in the aggregated state, making it effective in hypoxic tumor microenvironments. The molecular design of PdL, by increasing drug loading capacity and the stability of nanoparticles, ensures that the excited state energy is fully utilized under light conditions, thereby enhancing its efficacy in imaging and therapeutic applications.

## CONCLUSION AND OUTLOOK

Phototheranostic agents are indispensable tools in modern biomedicine, and their molecular design and performance optimization play a crucial role in advancing technological progress and application transformation. Classic PSs, such as porphyrins and phthalocyanines, have laid an important foundation for PDT; however, their application in deep tissue treatment is limited by light absorption range and tissue penetration. By introducing innovative molecular design strategies, recent developments in novel phototheranostic agents (such as porphyrinoids, cyanine dyes, and organic semiconductor polymers) have achieved significant breakthroughs in optical performance, multifunctionality, and stability, demonstrating great application potential and providing new possibilities for the construction of integrated diagnostic and therapeutic platforms.

Excited state regulation is one of the cores of the phototheranostic field. By precisely adjusting the optical performance and excited state behavior of PSs through strategies such as molecular design, coordination chemistry, and molecular self-assembly, therapeutic efficiency can be significantly enhanced. Specifically, optimizing the molecular chemical structure, metal-ligand interactions, and aggregation behavior can balance the energy dissipation pathways of PSs, and improve the stability and functional performance of excited states, thereby meeting the increasingly precise and personalized medical needs.

Despite significant advancements, phototheranostic agents face several limitations that must be addressed. Potential toxicity, particularly from the long-term accumulation of non-biodegradable PSs, poses a serious concern. Additionally, limited tissue penetration due to the absorption and scattering of light in biological tissues restricts the application of phototheranostics to superficial lesions or requires the use of near-infrared light for deeper penetration. Challenges in clinical translation, including regulatory hurdles and variability in patient response, also hinder broader adoption.

To overcome these challenges, future research should focus on the development of biodegradable PSs to minimize toxicity and ensure safe metabolism. The integration of machine learning algorithms for molecular design can accelerate the discovery of new PSs with optimized properties. Furthermore, innovative strategies for targeted delivery are essential for improving therapeutic efficacy while reducing side effects. For example, combining nanotechnology with targeted molecules such as antibodies or peptides can enhance specificity and reduce damage to healthy tissues.

Moreover, exploring synergistic treatments by integrating phototheranostics with other therapeutic modalities, such as immunotherapy, chemotherapy, or gene therapy, could unlock new avenues for enhancing treatment outcomes. Machine learning can further aid in predicting optimal combinations and personalizing treatment plans based on individual patient profiles. These advancements, coupled with interdisciplinary collaboration, promise to propel phototheranostics toward more effective, personalized, and broadly applicable therapies.

## Conflict of interest

Peipei Xing, Xinyue Wu and Mengliang Zhu declare that they have no conflict of interest.
